# The osteoporotic fracture prevention program in rural areas (OFRA): a protocol for a cluster-randomized health care fund driven intervention in a routine health care setting

**DOI:** 10.1186/s12891-016-1308-0

**Published:** 2016-11-08

**Authors:** Kilian Rapp, Karin Kampe, Patrick Roigk, Hella Kircheisen, Clemens Becker, Ivonne Lindlbauer, Hans-Helmut König, Dietrich Rothenbacher, Gisela Büchele

**Affiliations:** 1 Department of Clinical Gerontology, Robert-Bosch-Hospital, Auerbachstr. 110, 70376 Stuttgart, Germany; 2 Department of Medical Sociology and Health Economics and Health Services Research, University Medical Center Hamburg-Eppendorf, Martinistr. 52, 20246 Hamburg, Germany; 3 Department of Health Economics and Health Services Research, University Medical Center Hamburg-Eppendorf, Martinistr. 52, 20246 Hamburg, Germany; 4 Institute of Epidemiology and Medical Biometry, Ulm University, Helmholtzstr. 22, 89081 Ulm, Germany

**Keywords:** Accidental falls, Fractures, Osteoporosis, Preventive health services, Cluster-randomized

## Abstract

**Background:**

Fragility fractures are one of the leading causes for disability in old people. The main underlying mechanisms are osteoporosis and falls. Evidence-based measures to prevent either falls or fractures are available. However, coordinated preventive approaches combining bone health and fall prevention are rare.

The objective of the study is to evaluate a health care fund driven program, which encourages insured persons to adhere to national guidelines regarding bone health and physical activity and falls prevention. The health care fund cooperates with the ‘German Association of Rural Women’ and the ‘German Gymnastics Association’. The program consists of mobility and falls prevention classes, the examination of bone health by a DXA scan, and a consultation about ‘safety in the living environment’.

**Methods:**

Cluster-randomized study in 47 intervention and 143 control districts in 5 federal states of Germany. The program is offered to a) community-living women and men aged 70 to <85 years with a prior fragility fracture or b) community-living women aged 75 to <80 years. Within two years more than 10,000 persons will be directly contacted and motivated to make use of the components of the program. The primary outcome is a combined measure of incident osteoporotic fractures. Secondary outcomes include the rate of referrals to a mobility and falls prevention class or a bone mass density measurement. An economic evaluation will be conducted.

**Discussion:**

The study evaluates a complex preventive intervention in a routine health care setting which may serve as model for similar approaches in other areas or countries.

**Trial registration:**

DRKS-ID: DRKS00009000; date of registration: 06.08.2015

## Background

Falls and fall-related fractures are a major health problem in aging societies undergoing demographic transition [1]. Functional impairment, psychological problems like anxiety or depression and the disruption of the personal social infrastructure are frequent and amongst the primary reasons for disability and loss of autonomy in older people. Femoral fractures are the most common, costly, and resource consuming type of fragility fractures [2]. The secular trend in rates of femoral fractures is inconsistent. While some countries reported a decline over the last decade, other countries such as Germany found unchanged rates [3, 4]. Due to the growing number of old and very old persons in industrialized countries, the absolute number of fractures is expected to rise substantially over the coming years. In Germany, for example, the absolute number of fractures attributable to osteoporosis will increase between 2010 and 2050 by 238 % if fracture rates remain constant [2]. But even without the occurrence of fall-related injuries a pure functional decline with loss of capabilities like outdoor mobility or stair climbing may threaten older people’s independence. Therefore, there is an urgent need for measures and strategies which stop or even reverse to some extent older people’s functional decline and prevent falls and fall-related fractures.

The two main underlying mechanisms of fragility fracture are osteoporosis and falls [5]. For the prevention of falls, various strategies have been shown to be effective [6]. For community-living people, there is a large body of evidence that physical exercise with components of strength and balance training is the most effective measure [6]. Furthermore, it has been shown that physical exercise does not only prevent falls but also fractures [7]. These effects are mediated by measurable improvements in muscle strength and balance [8] which are of importance not only for safe walking but also for the performance of most of the basic activities of daily living. However, not all falls are preventable. An additional approach is to diminish the consequences of falls. Several pharmaceutical agents offer effective treatment options for the improvement of bone quality. Randomized controlled trials found risk reductions of new fragility fractures by pharmaceutical therapies of 30-50 % [9, 10]. These treatment effects are impressive but mainly restricted to people with osteoporosis which account only for a limited age-specific percentage of old people with an increased fracture risk. Therefore, coordinated preventive approaches are needed which combine bone health and fall prevention in order to reduce the burden of falls and fall-related fractures [11].

In most western countries fall prevention offerings for community-living people are limited and not well coordinated. The availability of exercise classes in rural areas may be particularly problematic. People from these areas are usually confronted with long and unacceptable distances if they want to attend an exercise class. Furthermore, bone health is a neglected field in many countries in the world and only few patients with fragility fractures receive osteoporosis investigation or treatment [12, 13]. Part of the problem is that the awareness of osteoporosis and of the potential treatment options is still low [14]. To improve health care in patients with fragility fractures particularly Anglo-Saxon and Scandinavian countries started to implement disease-management programs for secondary prevention of fractures. Most of the programs focus on the diagnosis and treatment of osteoporosis and use specialized persons (‘care manager’, ‘fracture liaison nurses’) to approach and supervise patients. These programs differ due to different included components, different intensities, and different settings [14, 15]. The so far most comprehensive approach was performed by a health maintenance organization in Southern California which approached their insured persons at risk actively and addressed not only bone health but also fall risk [16]. All these programs, however, had either only historical control groups or evaluated only process variables like DXA measurements or medical therapy [15]. Coordinated preventive approaches which combine bone health and fall prevention are rare. In Germany such approaches do not exist at all. Even though bone density measurement for primary prevention is generally recommended by the German national guidelines in women above 70 years and in men above 80 years (http://www.dv-osteologie.org/dvo_leitlinien/osteoporose-leitlinie-2014), it is reimbursed only in few cases and therefore performed rarely.

The objective of this study protocol is to present the idea, design and evaluation strategy of a large health care fund driven program conducted in Germany. This program is delivered in rural areas and wants to improve physical function and reduce the risk of falls and fractures in old people. The components of the program are mobility and falls prevention classes, the examination of bone health by a DXA scan, and a consultation about ‘safety in the living environment’.

## Methods / design

The osteoporotic fracture prevention program in rural areas (OFRA – German name of the program: ‘Trittsicher durchs Leben‘) is a large health care fund driven program to improve safe mobility and reduce the risk of falls and fractures in older people living in rural areas. The health care fund (Sozialversicherung für Landwirtschaft, Forsten und Gartenbau - SVLFG) is both a health insurance and an accident insurance which is compulsory for people working in agriculture, gardening and forestry. For OFRA the health care fund cooperates with the ‘German Association of Rural Women’ (LandFrauenverband - dlv), a volunteer organisation of women living in rural areas, the German Gymnastics Association (Deutscher Turnerbund - DTB) and the Robert Bosch Gesellschaft für Medizinische Forschung, Stuttgart (RBMF).

### Intervention areas / Randomisation

**Fig. 1:**
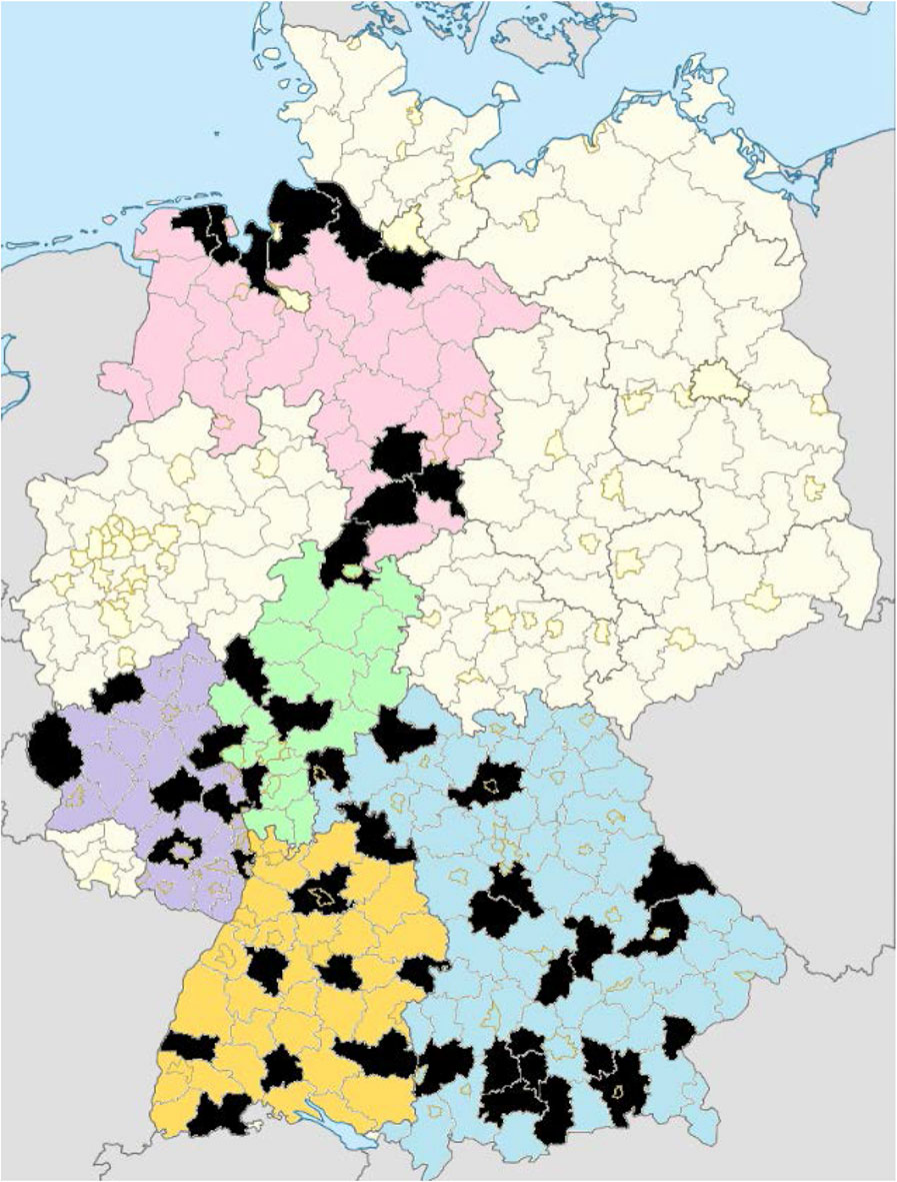
Map of Germany with 47 intervention districts (*black*) in the federal states Baden-Württemberg (*orange*), Bavaria (*blue*), Hesse (*green*), Lower Saxony (*pink*), and Rhineland-Palatinate (*purple*). The template of the map was derived from the public domain of Wikimedia Commons and modified by the authors from the file (https://commons.wikimedia.org/wiki/File:Landkreise,_Kreise_und_kreisfreie_St%C3%A4dte_in_Deutschland_2011-09-04.svg), which has been made freely available by Wikimedia User TUBS (2011)

The implementation of the program takes place in 47 administrative districts in the five federal states Baden-Württemberg, Bavaria, Hesse, Lower Saxony, and Rhineland-Palatinate which cover a large percentage of the whole area of Germany (Fig. 1 and Table 1). All administrative districts of the five federal states were randomly assigned to either the implementation or control group (1:3 cluster randomization; 47 intervention districts and 143 control districts). The control districts receive ‘usual care’.

**Table 1:** List of the implementation districts in the five federal states Baden-Württemberg, Bavaria, Hesse, Lower Saxony, and Rhineland-Palatinate

Federal state	Administrative districts
Baden-Württemberg	Biberach, Calw, Emmendingen, Esslingen, Heidenheim, Heilbronn, Main-Tauber-Kreis, Tuttlingen, Waldshut
Bavaria	Altötting, Aschaffenburg, Bad Kissingen, Bad Tölz-Wolfratshausen, Bamberg, Cham, Ebersberg, Fürstenfeldbruck, Kelheim, Landsberg am Lech, Pfaffenhofen a.d. Ilm, Rosenheim, Roth, Starnberg, Straubing-Bogen, Unterallgäu, Weilheim-Schongau, Weißenburg-Gunzenhausen
Hesse	Groß-Gerau, Kassel, Lahn-Dill-Kreis, Rheingau-Taunus-Kreis, Wetteraukreis
Lower Saxony	Cuxhaven, Friesland, Goslar, Harburg, Hildesheim, Northeim, Stade, Wesermarsch, Wittmund
Rhineland-Palatinate	Ahrweiler, Alzey-Worms, Bad Kreuznach, Bitburg-Prüm, Eifelkreis, Kaiserslautern, Rhein-Pfalz-Kreis

### Participants

The program is offered to a) all community-living women and men aged 70 to <85 years with a fragility fracture in the last 5 years and b) all community-living women aged 75 to <80 years who live in the intervention districts and are insured by the health care fund within the SVLFG (complete sampling). Insured people are excluded if they are institutionalized in a nursing home or if their care need for the basic activities of daily living is 120 minutes or more according to the categorization of the German long term care insurance (level of care: grade 2 or 3). The recruitment period started on 1st October 2015. Within two years more than 10,000 potential participants will be directly contacted and motivated to make use of one or more components of the program.

### Intervention program

OFRA (‘Trittsicher’) consists of 3 different components: 1.) ‘Trittsicher’-mobility and falls prevention classes, 2.) the examination of bone health by a DXA scan, and 3.) a consultation about ‘safety in the living environment’.

Ad 1.) ‘Trittsicher’-mobility and fall prevention classes are based both on the Otago exercise program [17, 18] and a falls prevention program developed for groups by the German Gymnastics Association [19]. Six sessions à 90 minutes are delivered within a time period of 6 weeks. Additionally to the group exercise classes, participants are advised to perform exercises at home between and after the end of the group sessions. Participants receive a booklet with instructions for the home training and a training log. The relevance of home training is stressed in every session and motivating factors and barriers are regularly discussed.

The classes are usually organized by active members of the ‘German Association of Rural Women’ (so-called LandFrauen) and take place in the countryside in order to keep the distances as short as possible for the participants who are often frail and have functional limitations. As a consequence, the places where the classes are offered are dependent from local conditions and can be parish halls, fire stations or even an adjoining room of a pub. The trainers are physiotherapists or exercise instructors from the local sports clubs who have got an additional education in the ‘Trittsicher’-program. The classes are usually free of charge and also open to older people who are not part of the scientific OFRA-program.

Ad 2.) A DXA scan for a bone density measurement is recommended to all included participants. The recommendation is based on the German osteoporosis guidelines of the Dachverband Osteologie (dvo) (http://www.dv-osteologie.org/dvo_leitlinien/osteoporose-leitlinie-2014). The bone density measurement (DXA) is reimbursed by the program independently if the participant has a fracture history or not. The participants are asked to talk with their general practitioners about the need of a DXA scan. In this case, the general practitioners receive a one-time payment for their counseling and for handling the potential consequences of the examination such as the treatment of a diagnosed osteoporosis.

Ad 3.) Nearly all participants are visited at home by a ‘prevention manager’ of the agricultural accident insurance (part of the SVLFG) and advised how to increase safety in the nearby living environment. Examples are the installation of handrails in the entrance area or better lighting on those paths around the farm which are still used by the participants.

**Fig. 2:**
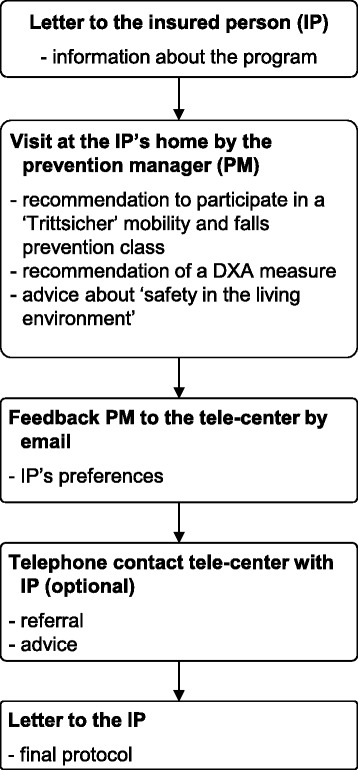
Intervention scheme

The selected persons in the intervention districts are informed about the new program by a personal letter (Fig. 2). One to four weeks later a ‘prevention manager’ visits the insured persons at home and motivates them to attend a ‘Trittsicher’-mobility and fall prevention class or to make use of a DXA measurement. If required, they will also give recommendations how to increase safety in the nearby living environment. In few cases the approach can be also by telephone instead of a visit at home. The ‘prevention managers’ are usually well known by the insured persons since they visit the farms regularly as part of their daily work for the agricultural accident insurance. The ‘prevention manager’ gives feedback about the insured person’s preferences to the tele-center. In case of interest the staff of the tele-center tries to refer the insured person to one of the ‘Trittsicher’-mobility and falls prevention classes taking place next to the people’s homes. The persons receive a final report in which their preferences regarding the components of the program, dates and arrangements are summarized. The report includes also information sheets for the general practitioner and the medical specialist with the DXA scanner with the recommendation of a bone density measurement and details about reimbursement (Fig. 2).

### Prearrangements

Germany has a general healthcare gap in the supply of mobility and fall prevention classes which is particularly pronounced in rural areas. Therefore, one of the objectives of OFRA is to implement an infrastructure of ‘Trittsicher’-exercise instructors and of mobility and falls prevention classes. In the year before the start of recruitment about 700 exercise instructors and physiotherapists from intervention districts received a mandatory one-day ‘Trittsicher’-training course. In addition, more than 500 ‘LandFrauen’ agreed to generally organize ‘Trittsicher’ mobility and fall prevention classes in the countryside after having been informed about the program in their local meetings. Three tele-centers were set up in towns of three different federal states. The staff of the tele-centers supports the organization of mobility and falls prevention classes, arranges participants’ referrals to the classes and serves as contact persons for the involved groups. The SVLFG and the Association of Statutory Health Insurance Physicians of the participating federal states closed a contract regulating the reimbursement of the general practitioners and of the bone density measurement. A website serves as information platform for the public and for the involved groups (www.trittsicher.org).

### Evaluation

OFRA is a complex intervention which is offered as a cluster-randomised large simple trial to the 47 intervention districts in 5 federal states of Germany. The implementation of the program is based on evidence-based guidelines and is conducted in an existing health care delivery system. The effect of the delivery of the guidelines is the matter of interest. The evaluation sticks to the principles of implementation research and considers not only outcome research but also the effect of the program on structures and processes [20, 21]. Furthermore, a cost-effectiveness analysis will be carried out one year after the end of recruitment.

### Outcome measures

The primary outcome is a combined measure of incident osteoporotic fractures (ICD-10 S12, S22, S32, S42, S52, S72, S82, combined) requiring hospitalization. Data derive from the routine health claims database of the SVLFG and will be extracted one (and two) years after the end of the recruitment period.

Secondary outcomes will be the attendance of a ‘Trittsicher’-mobility and falls prevention class, the referral to a bone mass density measurement (DXA) and a new antiresorptive drug therapy for osteoporosis (e.g. bisphosphonates) eight months after the first contact. In addition, the participants’ motivational factors, barriers, and beliefs will be assessed. The secondary outcomes will be obtained by telephone interview or by using routine health claims data in a randomly selected subgroup of 500 participants. In addition, the Trittsicher’-mobility and falls prevention classes and the number and characteristics of their participants are continuously monitored.

### Sample size and statistics

All eligible persons in the intervention districts will be allocated to the program and those with the same properties in the control districts serve as controls. The expected fracture rate (composite endpoint) in the control group is 30/1000 person-years. For an assumed reduction of 20 % a sample size of 9,814 persons in the intervention districts and 29,441 persons in the control districts during a mean follow-up time of 24 months is necessary by additionally considering an intra-class correlation.

To analyze the effect of the program on the fracture rate a poisson regression analysis will be applied. The cluster structure will be considered by a multi-level analysis. Adjustment by individual or structural variables will be considered, if necessary.

Based on routine data of the health care fund fracture-related costs of acute care, rehabilitation, and nursing care, as well as expenses for bone mass density measurement and drug therapy for osteoporosis will be calculated in the intervention and control group. Implementation costs will be calculated based on accounting principles taking into account protocol costs as well as information collected from prevention advisors. The primary health effect is the combined measure of incident osteoporotic fracture requiring hospitalization (see above). The incremental cost-effectiveness (ICER) will be calculated over the whole observation period and also the net-benefit approach will be applied to control for possible confounding factors in ICER [22].

All analyses will be performed with anonymized datasets. Participants are asked to give an informed consent in case they want their personal data to be passed on for example to the organizer of a ‘Trittsicher’-mobility and falls prevention class.

The evaluation will be performed by the Department of Clinical Gerontology, Robert-Bosch-Hospital, Stuttgart in cooperation with the Institute of Epidemiology and Medical Biometry, Ulm University and the Department of Health Economics and Health Services Research, University Medical Center Hamburg-Eppendorf. The evaluation of the program was registered at the German Clinical Trials Register (https://drks-neu.uniklinik-freiburg.de/drks_web/) (DRKS-ID: DRKS00009000) and approved by the ethics committee of Ulm University (proposal 120/15).

## Discussion

The study analyzes a health care fund driven innovative health service approach. The program wants to improve safe mobility and reduce fragility fractures in old people in rural areas. Insured persons with an increased risk for falls and fractures will be supported in adopting and realising already existing evidence-based guidelines concerning bone health and physical exercise/fall prevention. These are so far neglected topics in the German health care system even though evidence-based measures are available. Therefore, OFRA addresses an important implementation gap.

The study has a high sample size and the intervention is embedded in a broad setting. The structural difficulties of rural areas are compensated by fostering the cooperation between organizations which are available and influential in rural areas. The case finding strategy in the area of falls prevention and bone health is an innovative approach in the German health care system. The combination with ‘prevention managers’ visiting the people at home may be unique even worldwide.

The program has also limitations and may be confronted with program-specific problems. The organization and implementation of ‘Trittsicher’-mobility and falls prevention classes will need a high input of personal resources until an infrastructure of classes is implemented. The next step in the implementation process has to be the transformation of single classes into permanent offerings. Therefore, it will be crucial that the organizing ‘LandFrauen’, the exercise instructors and the participants all experience the classes as a useful contribution to personal wellbeing and social life.

Participants will be motivated to perform a DXA measurement. The potential consequences of the diagnosis of osteoporosis are beyond the scope of the program. It is unrealistic to expect that OFRA will change general practitioners’ beliefs and practices regarding bone health within a short time period. Therefore, some of the participants may be not treated according to the guidelines even if an osteoporosis was diagnosed. Furthermore, DXA devices are rare in some rural regions and participants may not make use of a measurement since the distance to the next device is too far.

The ‘prevention managers’ give advice how to increase safety in the nearby living environment. It is only a recommendation and its realization is not mandatory. Therefore, it may be postponed or even ignored. However, the ‘prevention managers’ have a high reputation on the farms and their recommendations are usually taken seriously.

The study has a clear scientific design. To our knowledge, it is the first study which analyses the effect of a coordinated preventive approach on the incidence of osteoporotic fractures by a randomized study design. However, OFRA is not a clinical trial but a complex health care fund driven intervention in a routine health care setting. Therefore, many uncontrollable factors may have an influence on the processes and thus on the effectiveness of the program. To account for some of these factors an accompanying process evaluation will be performed. But the evaluation of important expected effects like the change of functional capacity or of social participation is beyond the means within a large simple trial. In addition, the power calculation was based on assumptions which were partly difficult to anticipate like the treatment rate of people diagnosed with an osteoporosis due to the program. Therefore, the large number of directly addressed people could be still too low to detect a statistical significant reduction of osteoporotic fractures.

The partners within the intervention districts will be actively supported in the implementation of ‘Trittsicher’-mobility and falls prevention classes. However, a few classes will be also implemented in control districts by the partners' own initiative. Therefore, contamination cannot be completely avoided.

The health insurance fund has a very well defined population and a specific structure. In addition, some of the partners are only active in rural areas. Therefore, the generalizability of the approach may be limited. But the implementation gap is similar for old people living in rather urban areas or insured in other health care funds. Therefore, if OFRA shows to be effective it may serve as model which could be implemented in a modified way also by other health care funds, in other areas or even in other countries.
